# Circ-UBR4 regulates the proliferation, migration, inflammation, and apoptosis in ox-LDL-induced vascular smooth muscle cells via miR-515-5p/IGF2 axis

**DOI:** 10.1515/med-2023-0751

**Published:** 2023-08-30

**Authors:** Liuliu Feng, Tianhua Liu, Jun Shi, Yu Wang, Yuya Yang, Wenyin Xiao, Yanyan Bai

**Affiliations:** Department of Cardiology, Shidong Hospital, 200438, Shanghai, China; Department of Cardiology, Shidong Hospital, No. 999 Shiguang Road, Yangpu District, 200438, Shanghai, China

**Keywords:** circ-UBR4, miR-515-5p, IGF2, ox-LDL, VSMCs

## Abstract

The aim of our study is to disclose the role and underlying molecular mechanisms of circular RNA ubiquitin protein ligase E3 component *n*-recognin 4 (circ-UBR4) in atherosclerosis (AS). Our data showed that circ-UBR4 expression was upregulated in AS patients and oxidized low-density lipoprotein (ox-LDL)-induced vascular smooth muscle cells (VSMCs) compared with healthy volunteer and untreated VSMCs. In addition, ox-LDL stimulated proliferation, migration, and inflammation but decreased apoptosis in VSMCs, which were overturned by the inhibition of circ-UBR4. miR-515-5p was sponged by circ-UBR4, and its inhibitor reversed the inhibitory effect of circ-UBR4 knockdown on proliferation, migration, and inflammation in ox-LDL-induced VSMCs. Insulin-like growth factor2 (IGF2) was a functional target of miR-515-5p, and overexpression of IGF2 reversed the suppressive effect of miR-515-5p on ox-LDL-stimulated VSMCs proliferation, migration, and inflammation. Collectively, circ-UBR4 knockdown decreased proliferation, migration, and inflammation but stimulated apoptosis in ox-LDL-induced VSMCs by targeting the miR-515-5p/IGF2 axis.

## 1 Introduction

Atherosclerosis (AS), an inflammatory disease, is a crucial pathogenic factor of most cardiovascular and cerebrovascular diseases, characterized by lipid metabolism disorder and chronic inflammation [1,2]. AS is a multistep disease and can be induced by various risk factors [3]. The dysfunction and inflammation of vascular smooth muscle cells (VSMCs) are closely related to AS progression [4], and oxidized low-density lipoprotein (ox-LDL)-induced VSMCs’ injury are often used to construct AS models *in vitro* [5,6]. Therefore, elucidation of the molecular mechanism affecting ox-LDL induced VSMCs injury is expected to provide potential molecular targets for the treatment of AS.

Circular RNAs (circRNAs) are novel group of endogenous non-coding RNAs with a circular structure generated by back-splicing [7]. Previous research confirmed that circRNAs played significant functions in AS progression and served as independent markers for AS diagnosis [8]. For example, the inhibition of circCHFR impeded the cell growth and mobility of VSMCs through microRNA (miRNA)-mRNA network [9]. Hsa_circ_0010283 is derived from the ubiquitin protein ligase E3 component *n*-recognin 4 (UBR4) gene, also named as circ-UBR4. Circ-UBR4 was initially manifested to be highly expressed in ox-LDL-treated VSMCs by circRNA microarray analysis [9], and the functional effects of circ-UBR4 on ox-LDL-treated VSMC activities were partly elucidated [10,11]. However, the regulatory mechanisms of circ-UBR4 in AS progression are complex and have not been fully uncovered. Therefore, circ-UBR4 was selected as a target in our study to explore its role and molecular mechanism in ox-LDL-induced VSMCs injury, providing more evidence for it to be a potential target in AS therapy.

miRNAs are non-coding and single-strand RNAs (18–25 nucleotides) and play vital roles in multiple pathological processes [12]. Mechanistically, miRNAs have been confirmed to function inhibitory effects on gene expression by targeting 3′untranslational region (UTR) of their target genes [13]. Abundant reports discovered that miR-515-5p acted as a tumor-suppressor in numerous tumors [14,15,16]. We guessed that miR-515-5p also played significant roles in AS progression.

Insulin-like growth factor2 (IGF2) is a member of insulin-like growth factor (IGF) family [17]. The potential association between IGF family and hematological malignancies was revealed in a previous report [18]. Interestingly, Sun et al. also revealed that ox-LDL-induced upregulation of IGF2 could activate downstream IGF-related pathways, thereby enhancing the proliferation of VSMCs [19]. Therefore, the roles and functional effects of IGF2 were explored in AS progression.

In this research, ox-LDL-stimulated VSMCs were used as cell models of AS. Through bioinformatics analysis, we found that circ-UBR4 had complementary binding sites for miR-515-5p, and miR-515-5p could target IGF2. Therefore, we determined the role of circ-UBR4 and investigated the interplays among circ-UBR4, miR-515-5p, and IGF2 in ox-LDL-induced VSMCs.

## 2 Materials and methods

### 2.1 Serum samples

The serum samples were collected from 31 AS patients and 25 healthy normal controls in Shanghai Shidong hospital. The written informed consent was acquired from each patient and volunteer, and our study was approved by the Ethics Committee of Shanghai Shidong hospital.

### 2.2 Cell culture

T/G HA-VSMCs (derived from Aorta) were purchased from ATCC (catalogue: PCS-100-012; Manassas, VA, USA) and cultured in Dulbecco’s modified eagle medium (GIBCO BRL, Grand Island, NY, USA) containing 10% (v/v) fetal bovine serum (FBS; Thermo Fisher Scientific, Carlsbad, CA, USA) at 37°C with 5% CO_2_. The medium was replaced for every 2 or 3 days. Cells between passages 3 and 6 were employed in this study. To establish cell models of AS, VSMCs were treated with ox-LDL (Thermo Fisher Scientific) with different concentrations for 24 h or 50 μg/mL for different times. In function experiments, VSMCs were treated with 50 μg/mL of ox-LDL for 24 h after transfection for 24 h.

### 2.3 Cell transfection

Short interfering RNA (siRNA) targeting circ-UBR4 (si-circ-UBR4), control (si-NC), circ-UBR4 overexpression vector (circ-UBR4), empty vector (pCD5-ciR), IGF2-overexpression vector (IGF2), and empty vector (pcDNA) were designed and optimized by GenePharma (Shanghai, China). MiR-515-5p mimic (miR-515-5p), miR-NC, miR-515-5p inhibitor (anti-miR-515-5p), and anti-miR-NC were synthesized by Sangon (Shanghai, China). For cell transfection, VSMCs were seeded into 24-well plates (5 × 10^4^ cells/well) and then transfected with plasmids (2 μg), miRNA oligonucleotides (50 nM), or siRNA (50 nM) by Lipofectamine 2000 reagent (Thermo Fisher Scientific) referring to the recommended protocol.

### 2.4 Real-time quantitative PCR (RT-qPCR)

Total RNAs were isolated by TRIzol reagent (Invitrogen, Carlsbad, CA, USA) in compliance with the manufacturer’s direction. After quantifying under the Nanodrop 2000c (Thermo Fisher Scientific), the extracted RNA was used to transcribe into complementary DNA (cDNA) by First-Strand cDNA Synthesis SuperMix (CapitalBio, Beijing, China). RT-qPCR reactions were performed by SYBR Green Real-Time PCR Master Mix (Thermo Fisher Scientific) under the Roche LightCycler (Roche, Basel, Switzerland). The comparative threshold cycle (Ct) method was carried out to evaluate the relative expression of target RNAs, with glyceraldehyde-3-phosphate dehydrogenase (GAPDH) or nuclear RNA U6 as the control. The primer sequences were as follows: circ-UBR4, 5′-AGTGTGGTTACAGCCAGCTC-3′ (forward) and 5′-ACCATAACTACCAGCGGCAC-3′ (reverse); UBR4, 5′-CCCCGGAACCAACTTCAGTC-3′ (forward) and 5′-TTGGCGGATTTCATCATTGCT-3′ (reverse); miR-515-5p, 5′-GCCGAGTTCTCCAAAAGAAAGC-3′ (forward) and 5′-CAGTGCAGGGTCCGAGGTAT-3′ (reverse); IGF2, 5′-ACGAAATATCCCGCCTCATTTAC-3′ (forward) and 5′-GCAGTTTCCGAGTCAGTGTTCA-3′ (reverse); U6, 5′-CTCGCTTCGGCAGCACA-3′ (forward) and 5′-AACGCTTCACGAATTTGCGT-3′ (reverse); and GAPDH, 5′-TGAACCATGAGAAGTATGAC-3′ (forward) and 5′-TCTTACTCCTTGGAGGCCA-3′ (reverse).

### 2.5 RNase R treatment and nuclear-cytoplasmic fractionation

Purified RNAs were incubated with RNase R (Epicentre Technologies, Madison, USA), followed by purification with Trizol (Invitrogen).

In addition, Cytoplasmic and Nuclear RNA Purification Kit was used for nuclear-cytoplasmic fractionation assay. Briefly, ice-cold lysis buffer J was added into culture plate to lyse VSMCs. After centrifuging, supernatant was collected as cytoplasmic RNA. The precipitate containing the nuclear RNA was incubated with buffer SK. Finally, cytoplasmic RNA and nuclear RNA were eluted with elution buffer E.

### 2.6 MTT assay

VSMCs were planted into 96-well plates (5,000 cells per well) and cultured at 37°C with 5% CO_2_. After incubation for 24 h, 20 μL of MTT solution (5 mg/mL; Thermo Fisher Scientific) was added into each well at 37°C, and cells were allowed to incubate for another 4 h. The formatted crystal formazan was dissolved by dimethyl sulfoxide (Thermo Fisher Scientific). Microplate reader was used to assess optical density value at 490 nm.

### 2.7 EdU assay

Cell proliferation was also evaluated by EdU incorporation assay using an EdU Apollo DNA *in vitro* kit (RiboBio, Guangzhou, China). Briefly, cells after transfection were incubated in 96-well plates (5,000 cells per well) for 24 h at 37°C and next subjected with 100 μL of 50 μM EdU per well. After culturing cells with EdU for 12 h, cells were fixed and then counterstained with DAPI. The EdU staining was observed via a fluorescence microscopy (Mshot, Guangdong, China).

### 2.8 Flow cytometry assay

Apoptosis of VSMCs was assessed by Annexin V-FITC Apoptosis Detection Kit (BD Pharmingen, Franklin Lakes, NJ, USA). After incubation for 24 h, transfected VSMCs were harvested as a single cell suspension (1 × 10^6^/mL) by trypsin digestion. The staining buffer containing Annexin V-FITC and propidium iodide was added to incubate VSMCs at 4°C for 30 min. The Flow Cytometer (Beckman Coulter, Miami, FL, USA) was used for apoptosis assay.

### 2.9 Transwell assay

For *in vitro* cell migration assay, 24-well transwell chamber (BD Pharmingen, San Jose, CA, USA) was used. VSMCs resuspended in 200 µL medium without FBS (5 × 10^4^ cells/well) were seeded into the upper compartment, while complete medium was used as nutrients to induce cell migration. After 24 h, the remaining culture medium and the cells that did not migrate were removed carefully, while the migrated cells were fixed and then stained with 0.1% crystal violet (Thermo Fisher Scientific). The migrated cells were imaged under a microscope (100× amplification; Mshot). The number of migrated cells was counted in five randomly selected regions.

### 2.10 Enzyme-linked immunosorbent assay (ELISA)

Cell culture supernatants were harvested by centrifugation at 1,000 × *g* for 10 min. The ELISA kit (Invitrogen; #BMS223INST; #BMS213HS; and #KAC1211) was used to detect the levels of tumor necrosis factor-α (TNF-α), Interleukin-6 (IL-6), and Interleukin-1β (IL-1β) according to the commodity instruction. The concentrations of TNF-α, IL-6, and IL-1β were obtained based on standard curve.

### 2.11 Western blot assay

Briefly, a protein extraction kit (Applygen Technologies, Beijing, China) was used to extract protein from VSMCs. Protein concentration was assessed by bicinchoninic acid protein assay (Applygen Technologies). 30 μg of protein was fractionated by sodium dodecyl sulfate-polyacrylamide gels and electroblotted onto nitrocellulose membranes (Bio-Rad, Hercules, CA, USA). The membranes were incubated in 4% skim milk solution and then reacted with antibodies at 4°C overnight, including anti-β-actin (ab8226, Abcam, Cambridge, MA, USA), anti-CyclinD1 (ab16633, Abcam), anti-matrix metallopeptidase 9 (MMP9; ab76003, Abcam), anti-BCL2-Associated X (Bax; ab325033, Abcam), anti-B-cell lymphoma-2 (Bcl-2; ab196495, Abcam), and anti-IGF2 (ab177467, Abcam). After washing with Tris-buffered saline with Tween 20, the membranes were incubated with HRP-conjugated secondary antibodies (Abcam). Finally, the Alpha Innotech Imaging System (ProteinSimple, Santa Clara, CA, USA) was used to visualize protein signal. The band density was analyzed using Image J software (NIH, Bethesda, MA, USA).

### 2.12 Dual-luciferase reporter assay

The downstream targets of circ-UBR4 and miR-515-5p were predicted by circRNA interactome (https://circinteractome.irp.nia.nih.gov/) and Starbase (http://starbase.sysu.edu.cn/), respectively. The circ-UBR4 sequences containing miR-515-5p binding sites were inserted into pmirGLO luciferase vector (GeneCreat, Wuhan, China), named as WT-circ-UBR4, with MUT-circ-UBR4 as control. Similarly, the wild- and mutant-type of IGF2 luciferase reporter vector (WT-IGF2 3′UTR and MUT-IGF2 3′UTR) were synthesized by GeneCreat. VSMCs were transfected with luciferase reporter vectors in the presence or absence of miR-515-5p by Lipofectamine 2000 (Thermo Fisher Scientific). Relative luciferase activity was assessed under the VICTOR2 fluorometry (PerkinElmer, Waltham, MA, USA) at 48 h post-transfection.

### 2.13 RNA immunoprecipitation (RIP) assay

RIP assay was carried out using Imprint^®^ RIP kit (Sigma, Louis, MO, USA) according to manufacturer’s instruction. For RIP assay, VSMCs were lysed in RIP lysis buffer, followed by incubation with magnetic beads embracing Ago2 (Millipore, Billerica, MA, USA) or IgG (Millipore) antibodies for 24 h at 4°C. The level of RNA enriched by RIP was assessed by RT-qPCR.

### 2.14 Pull-down assay

miR-515-5p probe and miR-NC probe were labeled with biotin by RiboBio (Bio-miR-515-5p and Bio-miR-NC). VSMCs were transfected with Bio-miR-515-5p and Bio-miR-NC and subsequently lysed by lysis reagent (Thermo Fisher Scientific). Cell lysates were cultured with Streptavidin-Dynabeads (Thermo Fisher Scientific). RNA complexes pulled down by Bio-miR-515-5p and Bio-miR-NC were captured by beads. RNA samples were washed from beads and analyzed by RT-qPCR.

### 2.15 Statistical analysis

For each experiment, we set three duplications in adjacent three wells of 96- or 24-well plates. A total of three independent experiments were performed at few days interval using the same batch of frozen cells at passages 3–6. All quantitative data were displayed as mean value ± standard deviation. SPSS 21.0 software (IBM, Somers, NY, USA) was used to process data and analyze differences. The statistical differences in different groups were analyzed by Student’s *t*-test or analysis of variance (followed by Tukey’s *post-hoc* test). *P*-value less than 0.05 was considered to be statistically significant.

## 3 Results

### 3.1 circ-UBR4 was overexpressed in ox-LDL-stimulated VSMCs

**Figure 1:**
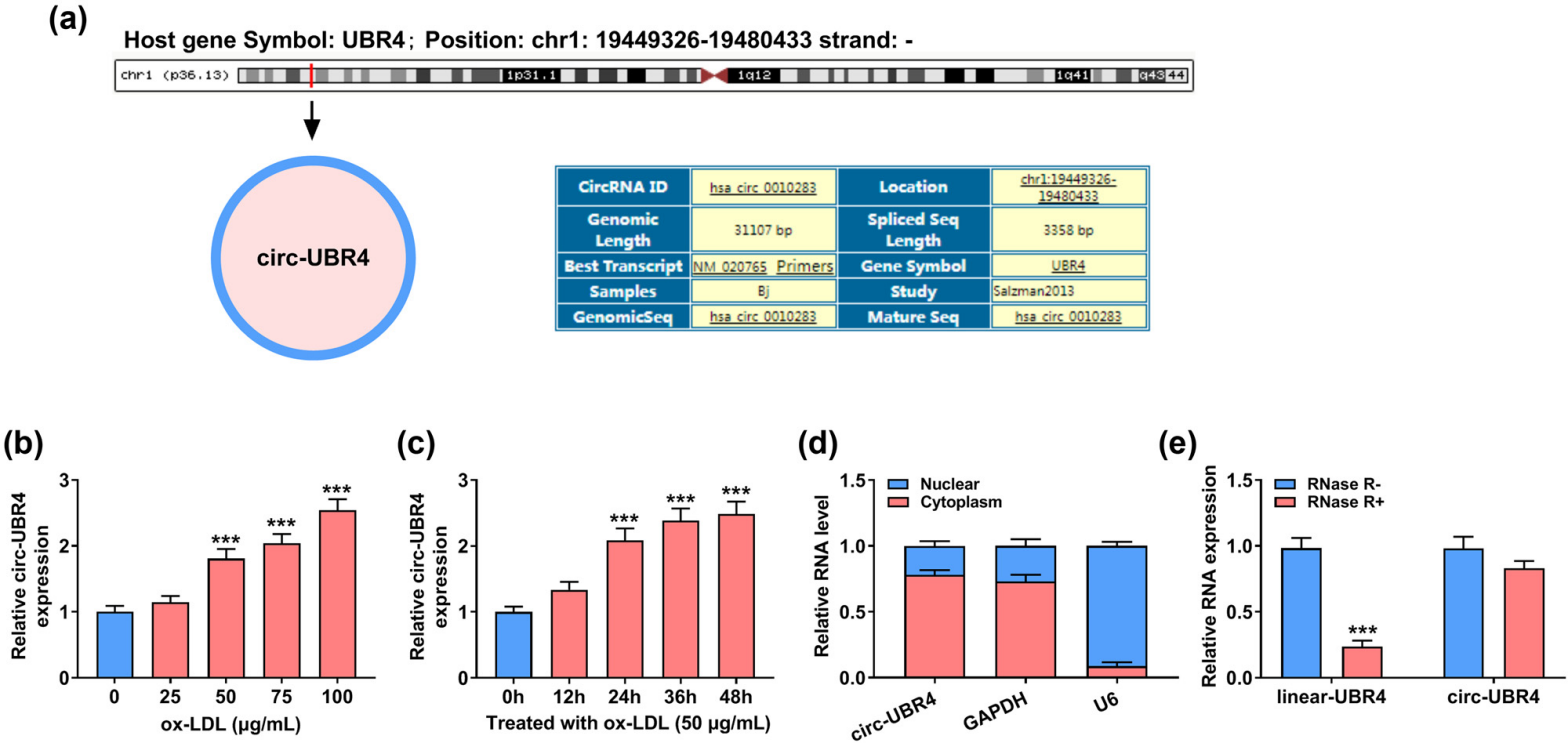
The expression level of circ-UBR4 in ox-LDL-stimulated VSMCs. (a) The information of circ-UBR4 structure and formation. (b) and (c) The relative expression level of circ-UBR4 was evaluated by RT-qPCR in ox-LDL-stimulated VSMCs. (d) The expression levels of circ-UBR4, GAPDH, and U6 were assessed by RT-qPCR in cytoplasmic and nuclear fraction RNAs. (e) RT-qPCR was used to show the expression of linear-UBR4 and circ-UBR4 in VSMCs after treatment with RNase R. ***P < 0.001.

**Figure A1:**
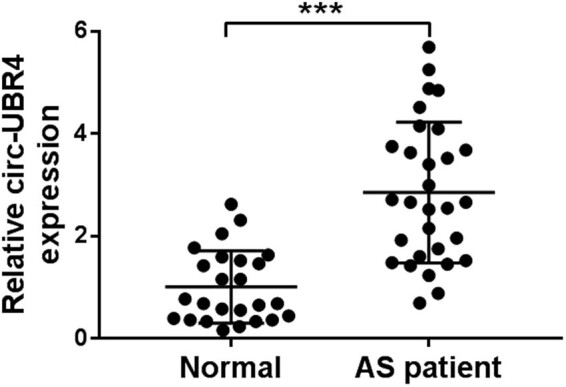
The expression of circ-UBR4 in the serum of AS patients or healthy normal controls. RT-qPCR was used to detect the expression of circ-UBR4 in the serum of AS patients (*n* = 31) or healthy normal controls (*n* = 25). ***P < 0.001.

Schematic diagram in Figure 1a manifested the information of circ-UBR4, showing that circ-UBR4 was produced from UBR4 gene (NM_020765), with 3358 bp in length. We detected circ-UBR4 expression in the serum of AS patients or healthy normal controls and confirmed that circ-UBR4 was overexpressed in AS patients (Figure A1). In order to analyze the potential role of circ-UBR4 in AS progression, we used ox-LDL-stimulated VSMCs as cell models of AS *in vitro*. As presented in Figure 1b, circ-UBR4 was significantly and dose-dependently increased in ox-LDL-stimulated VSMCs. Also, treatment with 50 μg/mL of ox-LDL significantly enhanced the expression of circ-UBR4 in VSMCs in a time-dependent manner (Figure 1c). RT-qPCR assay suggested the predominant cytoplasmic distribution of circ-UBR4 in VSMCs (Figure 1d). Furthermore, circ-UBR4 could resist the digestion of RNase R when compared with linear-UBR4 (Figure 1e). Therefore, the function of circ-UBR4 was explored in ox-LDL-stimulated VSMCs.

### 3.2 Knockdown of circ-UBR4 reversed ox-LDL-stimulated VSMCs functions

**Figure 2:**
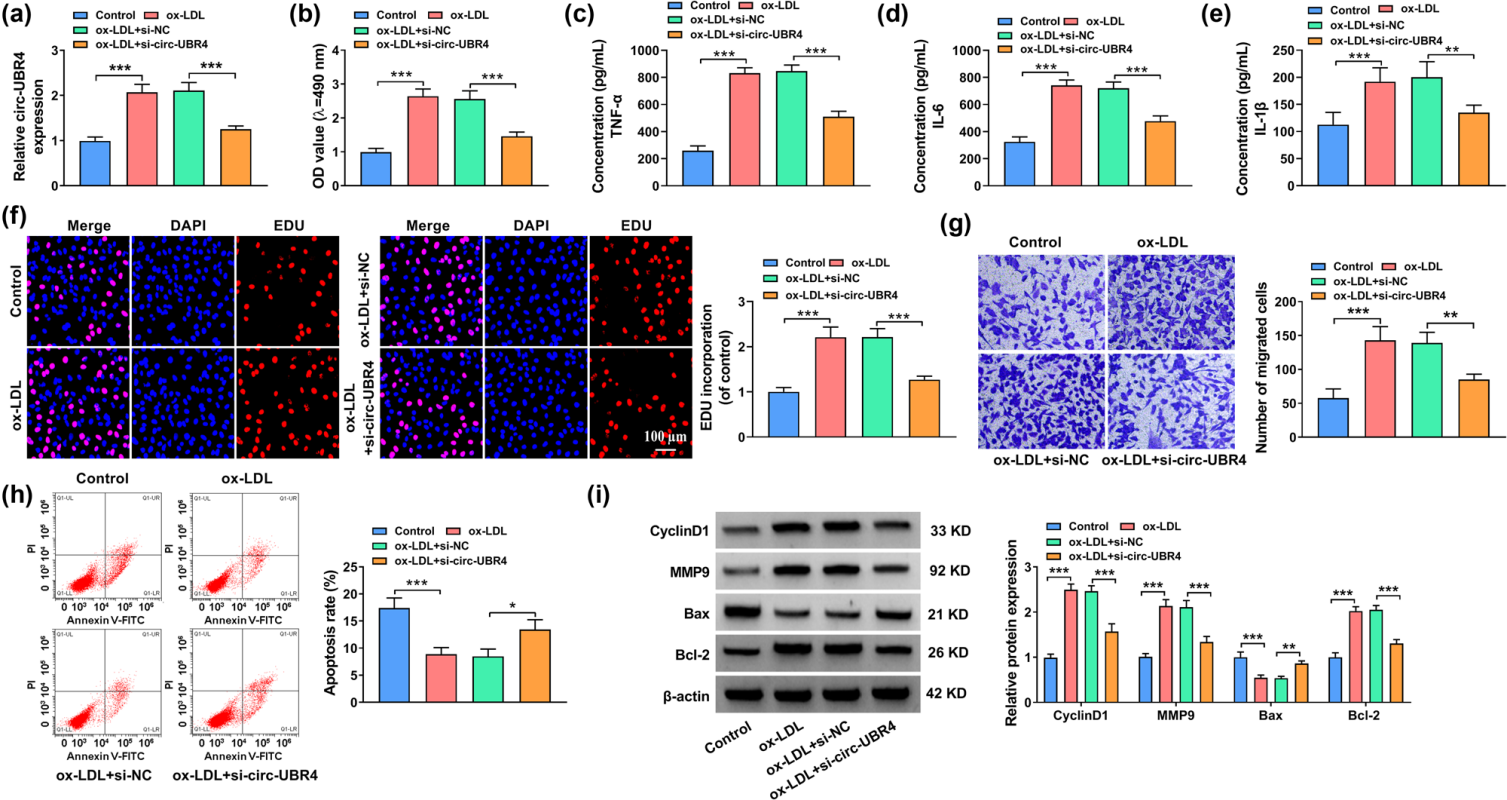
Inhibition of circ-UBR4 regulated proliferation, apoptosis, migration, and inflammation of ox-LDL-stimulated VSMCs. (a)–(i) VSMCs were divided into four groups: Control, ox-LDL, ox-LDL + si-NC, and ox-LDL + si-circ-UBR4. Un-treated VSMCs were used as Control. (a) The expression of circ-UBR4 was determined by RT-qPCR. (b) MTT assay was used for examining cell viability. (c)–(e) The levels of TNF-α, IL-6, and IL-1β were measured by ELISA. (f) EdU assay was used to examine cell proliferation. (g) Transwell assay was used to assess VSMCs migration. (h) The apoptosis of VSMCs was assessed by flow cytometry. (i) The protein expression levels of MMP9, CyclinD1, Bax, and Bcl-2 were quantified by western blot assay. *P < 0.05, **P < 0.01, ***P < 0.001.

As circ-UBR4 was overexpressed in ox-LDL-stimulated VSMCs, we next investigated the functional effects of circ-UBR4 inhibition in ox-LDL-stimulated VSMCs. Treatment with 50 μg/mL of ox-LDL enhanced the expression of circ-UBR4 in VSMCs, which was abolished by transfection with si-circ-UBR4 (Figure 2a). The results of MTT assay revealed that cell viability increased by ox-LDL was obviously inhibited by transfection with si-circ-UBR4 (Figure 2b). Similarly, knockdown of circ-UBR4 suppressed the inflammation in ox-LDL-stimulated VSMCs by reducing TNF-α, IL-6, and IL-1β levels (Figure 2c–e). Besides, ox-LDL-aggravated EdU incorporation and the number of migrated cells in VSMCs were largely repressed by circ-UBR4 downregulation (Figure 2f and g). Treatment with ox-LDL could inhibit cell apoptosis, while this effect was reversed by the knockdown of circ-UBR4 (Figure 2h). The results of western blot assay indicated that MMP9, CyclinD1, and Bcl-2 were upregulated, while Bax was downregulated in ox-LDL-stimulated VSMCs. However, the alterations induced by ox-LDL were all reversed by knockdown of circ-UBR4 (Figure 2i). Therefore, knockdown of circ-UBR4 inhibited cell proliferation, migration, and inflammation but promoted apoptosis in ox-LDL-stimulated VSMCs.

### 3.3 miR-515-5p was a direct target of circ-UBR4

**Figure 3:**
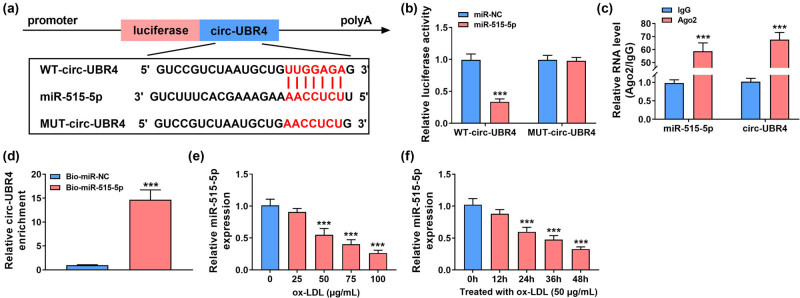
Circ-UBR4 regulated miR-515-5p expression in VSMCs. (a) Schematic diagram presented the complementary sequences between circ-UBR4 and miR-515-5p. (b)–(d) The potential association between circ-UBR4 and miR-515-5p was analyzed by dual-luciferase reporter, RIP, and pull-down assays. (e and f) RT-qPCR assay was performed to determine expression level of miR-515-5p in ox-LDL-stimulated VSMCs. ***P < 0.001.

**Figure A2:**
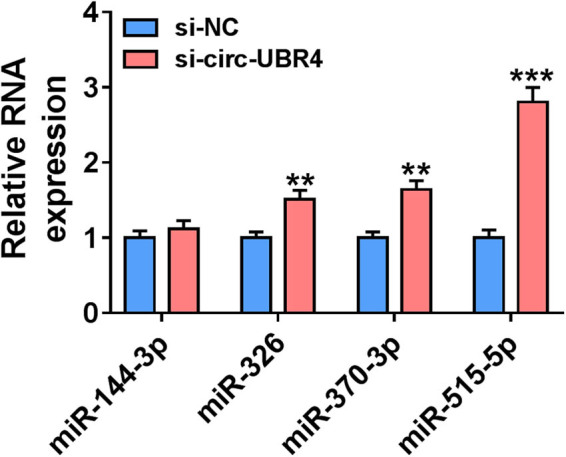
The selection of targeted miRNA of circ-UBR4. The expression of 4 miRNAs (miR-144-3p, miR-326, miR-370-3p and miR-515-5p) was detected by RT-qPCR in VSMCs transfected with si-circ-UBR4 or si-NC. **P < 0.01, ***P < 0.001.

The circRNA interactome software predicted that circ-UBR4 had more targeted miRNAs. Through literature research, four miRNAs (miR-144-3p, miR-326, miR-370-3p, and miR-515-5p) with low expression in AS and having an inhibition on proliferation and metastasis of VSMCs were selected for RT-qPCR analysis. The results show that circ-UBR4 knockdown could affect the expression of multiple miRNAs, but si-circ-UBR4 had the most obvious promotion effect on miR-515-5p expression (Figure A2). Therefore, miR-515-5p was selected as the target of circ-UBR4 for this study. The possible complementary sequences between circ-UBR4 and miR-515-5p are presented in Figure 3a. The upregulation of miR-515-5p led to great loss of luciferase activity in WT-circ-UBR4 group, while luciferase activity in MUT-circ-UBR4 group was not affected by miR-515-5p overexpression (Figure 3b). In RIP assay, the immunopurification of Ago2 can be performed to confirm the interaction between miRNA and target genes by detecting RNA enrichment. The results of RIP assay revealed that circ-UBR4 and miR-515-5p were all enriched by Ago2 (Figure 3c). Additionally, pull-down assay showed that high abundance of circ-UBR4 could be pulled down by Bio-miR-515-5p probe (Figure 3d). The above data confirmed the interaction between circ-UBR4 and miR-515-5p. MiR-515-5p expression was inhibited by ox-LDL in VSMCs in a dose-dependent manner and time-dependent manner (Figure 3e–f).

### 3.4 miR-515-5p inhibitor reversed the effect of circ-UBR4 knockdown on ox-LDL-induced VSMCs functions

**Figure 4:**
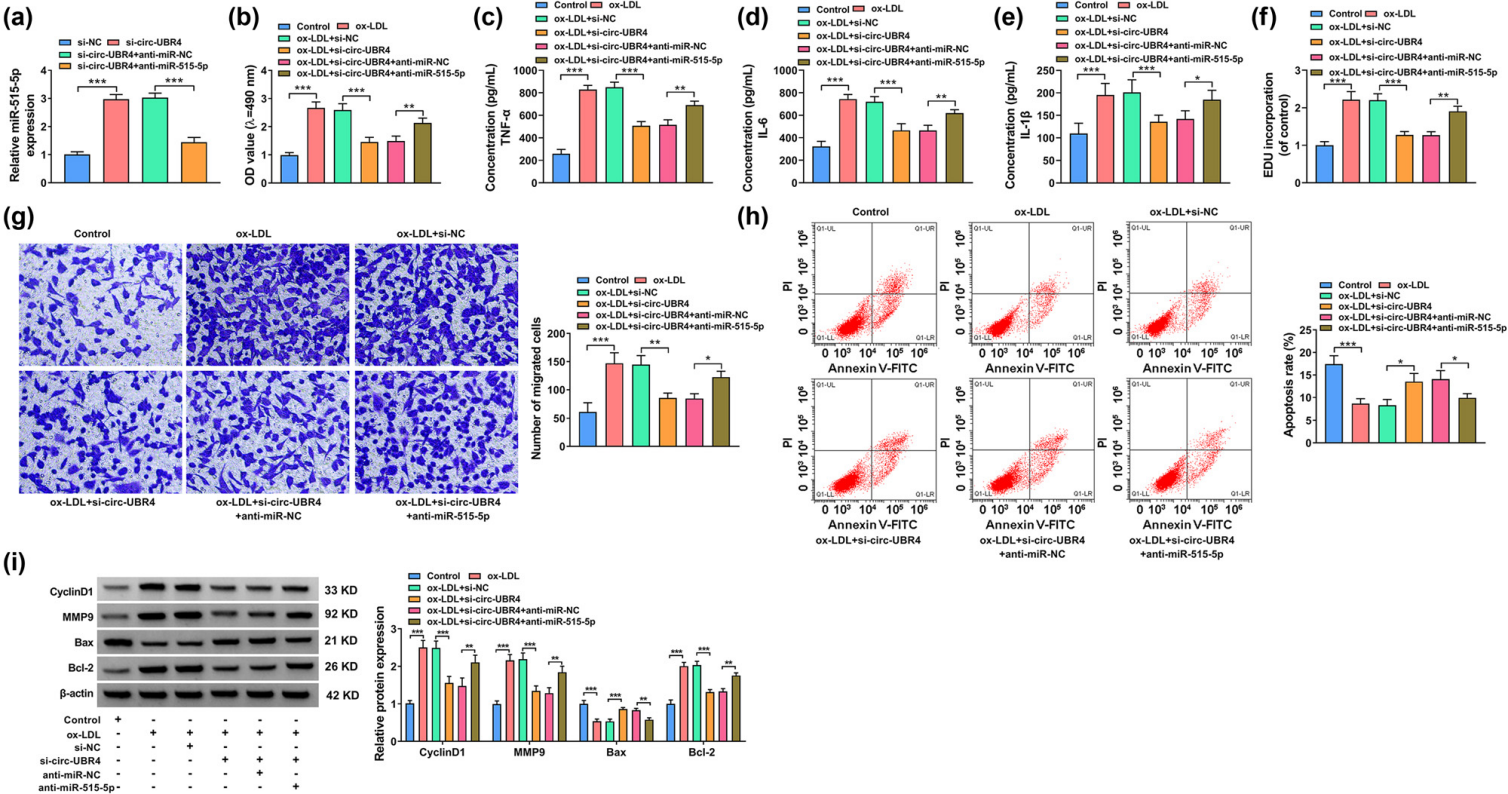
Knockdown of circ-UBR4-mediated effects in ox-LDL-stimulated VSMCs were abolished by silencing miR-515-5p. (a) RT-qPCR was performed to assess miR-515-5p level in VSMCs transfected with si-NC, si-circ-UBR4, si-circ-UBR4 + anti-miR-NC, or si-circ-UBR4 + anti-miR-515-5p. (b)–(i) VSMCs were divided into six groups: Control, ox-LDL, ox-LDL + si-NC, ox-LDL + si-circ-UBR4, ox-LDL + si-circ-UBR4 + anti-miR-NC, and ox-LDL + si-circ-UBR4 + anti-miR-515-5p. Untreated VSMCs were used as Control. (b) MTT assay was conducted to analyze cell viability. (c)–(e) The inflammation was assessed by measuring the levels of TNF-α, IL-6, and IL-1β by ELISA. (f) EdU assay was conducted to analyze cell proliferation. (g) The apoptosis of VSMCs was examined by flow cytometry assay. (h) The transwell was used to analyze VSMCs migration. (i) Western blot assay was performed to test protein expression levels of MMP9, CyclinD1, Bax, and Bcl-2 in VSMCs. *P < 0.05, **P < 0.01, ***P < 0.001.

**Figure A3:**
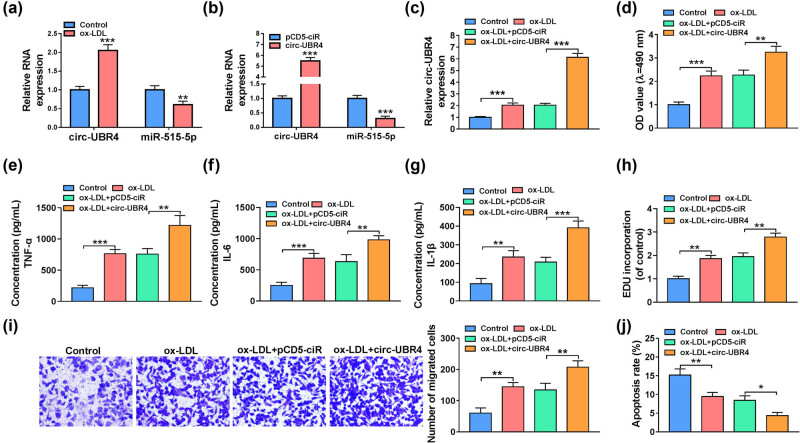
Effects of circ-UBR4 on miR-515-5p expression and ox-LDL-stimulated VSMCs function. (a) The expression levels of circ-UBR4 and miR-515-5p were assessed by RT-qPCR in VSMCs treated with or without ox-LDL. (b) The expression levels of circ-UBR4 and miR-515-5p were assessed by RT-qPCR in VSMSc transfected with pCD5-ciR or circ-UBR4 overexpression vector. (c–j) Ox-LDL-stimulated VSMCs were transfected with pCD5-ciR or circ-UBR4 overexpression vector, with un-treated cells as Control. (c) The circ-UBR4 expression was detected by RT-qPCR. (d) Cell viability was assessed by MTT assay. (e–g) ELISA was used to determine the levels of TNF-α, IL-6, and IL-1β. (h) Cell proliferation was assessed by EdU assay. (i) VSMCs migration was examined by transwell assay. (j) Cell apoptosis was examined by flow cytometry. *P < 0.05, **P < 0.01, ***P < 0.001.

The association between circ-UBR4 and miR-515-5p was investigated in ox-LDL-induced VSMCs. Transfection with anti-miR-515-5p abolished the upregulation of miR-515-5p expression in si-circ-UBR4-transfected VSMCs (Figure 4a). Inhibition of miR-515-5p could rescue the inhibition effect of si-circ-UBR4 on the viability, inflammation factor levels, proliferation, and migration of ox-LDL-treated VSMCs (Figure 4b–g). In addition, the downregulation of miR-515-5p protected VSMCs from si-circ-UBR4-induced apoptosis in ox-LDL-treated VSMCs (Figure 4h). MiR-515-5p knockdown also restored the expression of CyclinD1, MMP9, and Bcl-2 inhibited by circ-UBR4 silencing, and repressed the expression of Bax promoted by circ-UBR4 silencing in ox-LDL-treated VSMCs (Figure 4i). Therefore, the knockdown of miR-515-5p abolished circ-UBR4 inhibition-induced effects on ox-LDL-treated VSMCs. We measured the expression levels of both circ-UBR4 and miR-515-5p in the same ox-LDL-treated cells, and confirmed that circ-UBR4 was increased and miR-515-5p was decreased in ox-LDL-induced VSMCs (Figure A3a). In VSMCs transfected with circ-UBR4 overexpression vector, we found that circ-UBR4 was markedly promoted and miR-515-5p was significantly inhibited (Figure A3b). In addition, we examined cell proliferation, inflammation, migration, and apoptosis in ox-LDL-induced VSMCs-transfected circ-UBR4 overexpression vector. The circ-UBR4 expression was remarkably enhanced after the transfection of circ-UBR4 overexpression vector in ox-LDL-induced VSMCs (Figure A3c). Then, function experiments showed that circ-UBR4 overexpression promoted cell viability, the levels of inflammation factors (TNF-α, IL-6, and IL-1β), EdU incorporation, and the number of migrated cells, while suppressed apoptosis rate in ox-LDL-induced VSMCs (Figure A3d–j). These data confirmed that circ-UBR4 inhibited miR-515-5p expression to promote cell proliferation, migration, and inflammation, and inhibit apoptosis.

### 3.5 IGF2 was a functional gene of miR-515-5p

**Figure 5:**
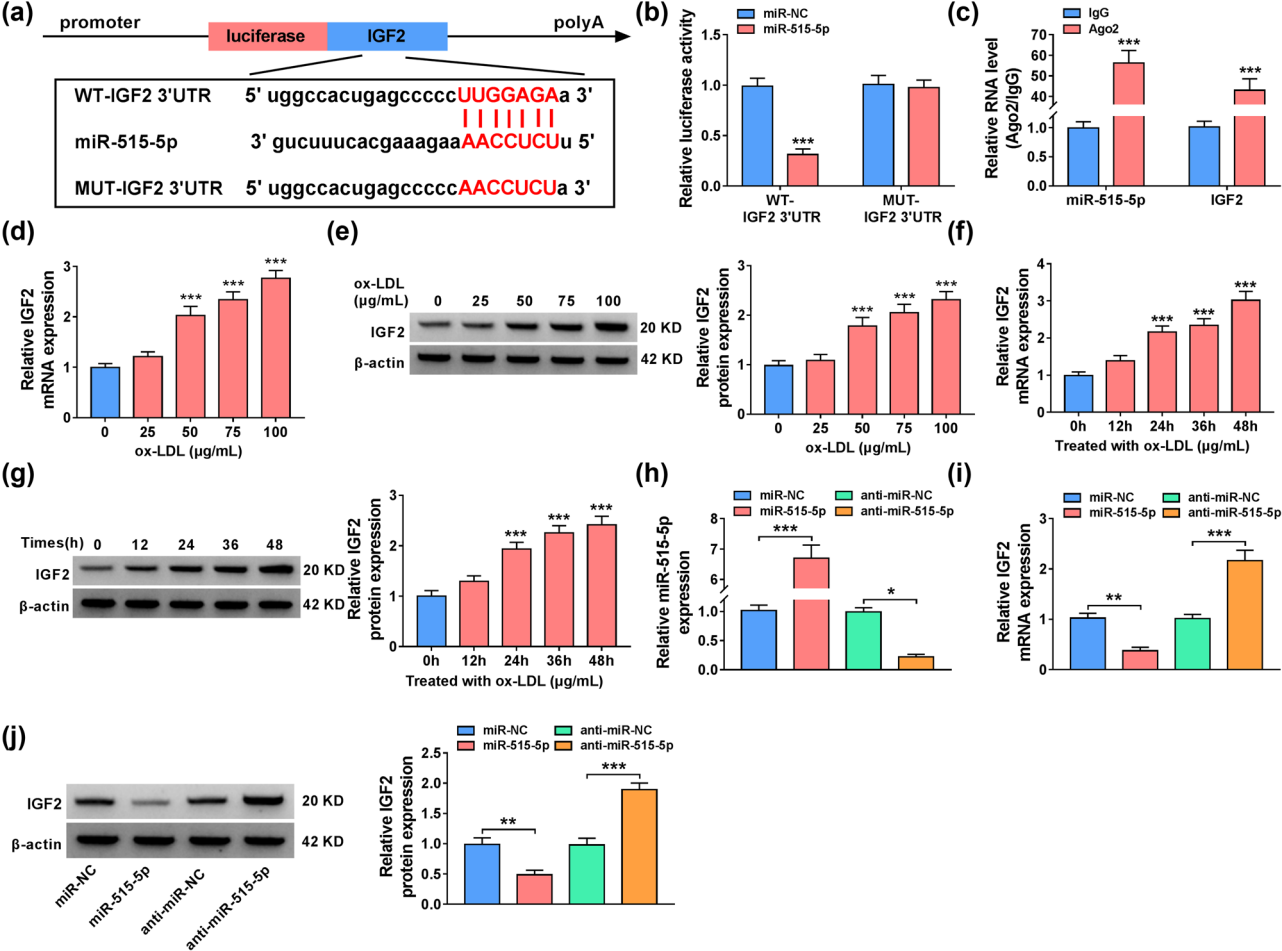
MiR-515-5p regulated the expression of IGF2 in VSMCs. (a) The binding regions between miR-515-5p and IGF2 were shown. (b) and (c) Dual-luciferase reporter and RIP assays were used to confirm the association between miR-515-5p and IGF2. (d)–(g) RT-qPCR and western blot assays were used to examine IGF2 levels in ox-LDL-stimulated VSMCs. (h) The expression level of miR-515-5p was assessed by RT-qPCR in VSMCs transfected with miR-NC, miR-515-5p, anti-miR-NC, or anti-miR-515-5p. (i) and (j) The mRNA and protein expression levels of IGF2 were measured by RT-qPCR and western blot assays in VSMCs transfected with miR-NC, miR-515-5p, anti-miR-NC, or anti-miR-515-5p. *P < 0.05, **P < 0.01, ***P < 0.001.

The online software Starbase was used to predict the target genes of miR-515-5p. We found that miR-515-5p had putative binding regions in 3′UTR of IGF2 mRNA (Figure 5a). Besides, the overexpression of miR-515-5p reduced the luciferase activity of WT-IGF2 3′UTR group but not MUT-IGF2 3′UTR group (Figure 5b). RIP assay also suggested that miR-515-5p and IGF2 were enriched in Ago2-immunoprecipitated complex when compared with control group, revealing the association between miR-515-5p and IGF2 (Figure 5c). Treatment with ox-LDL increased the mRNA and protein expression levels of IGF2 in VSMCs in dose-dependent manner and time-dependent manner (Figure 5d–g). Transfection with miR-515-5p mimic increased the expression of miR-515-5p in VSMCs, while miR-515-5p decreased in VSMCs after transfection with anti-miR-515-5p (Figure 5h). More importantly, IGF2 was substantially increased in the presence of anti-miR-515-5p in VSMCs but decreased in miR-515-5p-transfected cells, suggesting that IGF2 was negatively regulated by miR-515-5p (Figure 5i and j). These results together suggested that IGF2 was a direct target of miR-515-5p.

### 3.6 Overexpression of IGF2 reversed miR-515-5p-induced effects on ox-LDL-stimulated VSMCs functions

**Figure 6:**
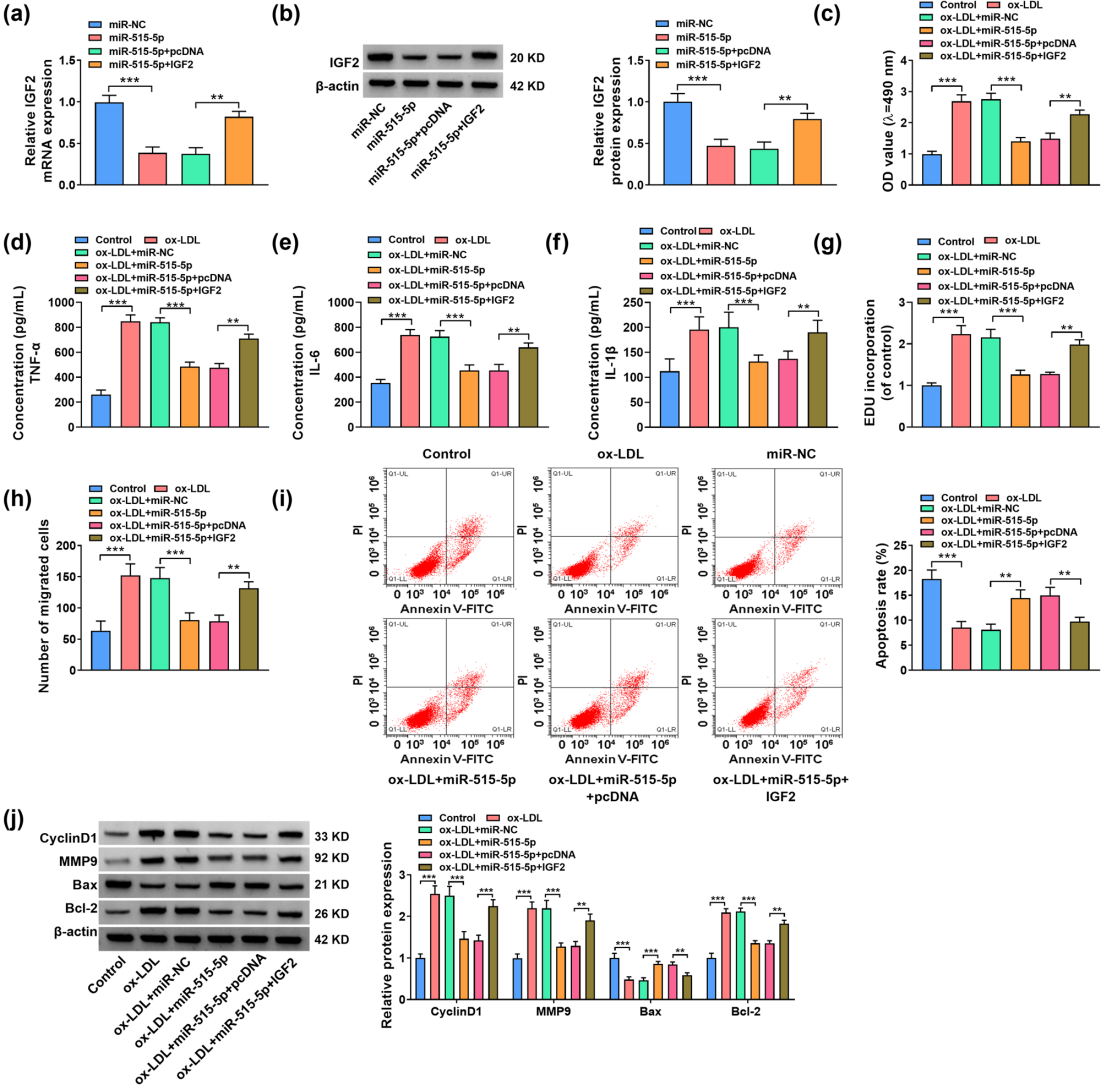
The miR-515-5p/IGF2 axis regulated proliferation, migration, inflammation, and apoptosis of ox-LDL-stimulated VSMCs. (a) and (b) The expression of IGF2 was assessed by RT-qPCR and western blot assays in VSMCs transfected with miR-NC, miR-515-5p, miR-515-5p + pcDNA, or miR-515-5p + IGF2. (c)–(j) ox-LDL-stimulated VSMCs were transfected with miR-NC, miR-515-5p, miR-515-5p + pcDNA, or miR-515-5p + IGF2, with untreated VSMCs as Control. (c) The cell viability of VSMCs was assessed by MTT assay. (d)–(f) ELISA was used to determine the levels of TNF-α, IL-6, and IL-1β in VSMCs. (g) The cell proliferation of VSMCs was assessed by EdU assay. (h) The migration of VSMCs was examined by transwell assay. (i) The apoptosis of VSMCs was examined by flow cytometry. (j) The protein expression levels of CyclinD1, MMP9, Bax, and Bcl-2 were assessed by western blot. *P < 0.05, **P < 0.01, ***P < 0.001.

The expression level of IGF2 was inhibited by the overexpression of miR-515-5p in ox-LDL-stimulated VSMCs, which was overturned by transfection with IGF2 (Figure 6a and b). The upregulation of miR-515-5p inhibited the viability, inflammation, proliferation, and migration in ox-LDL-stimulated VSMCs, while these effects were counteracted by the overexpression of IGF2 (Figure 6c–h). MiR-515-5p enrichment-triggered apoptosis of ox-LDL-treated VSMCs was largely inhibited by IGF2 overexpression (Figure 6i). Also, miR-515-5p decreased the Cyclin D1, MMP9, and Bcl-2 protein expression, while it enhanced the Bax protein expression in ox-LDL-stimulated VSMCs. However, these effects were reversed by significantly decreased IGF2 overexpression (Figure 6j). Above all, miR-515-5p regulated proliferation, migration, inflammation, and apoptosis in ox-LDL-stimulated VSMCs by targeting IGF2.

### 3.7 circ-UBR4 regulated IGF2 by targeting miR-515-5p

**Figure 7:**
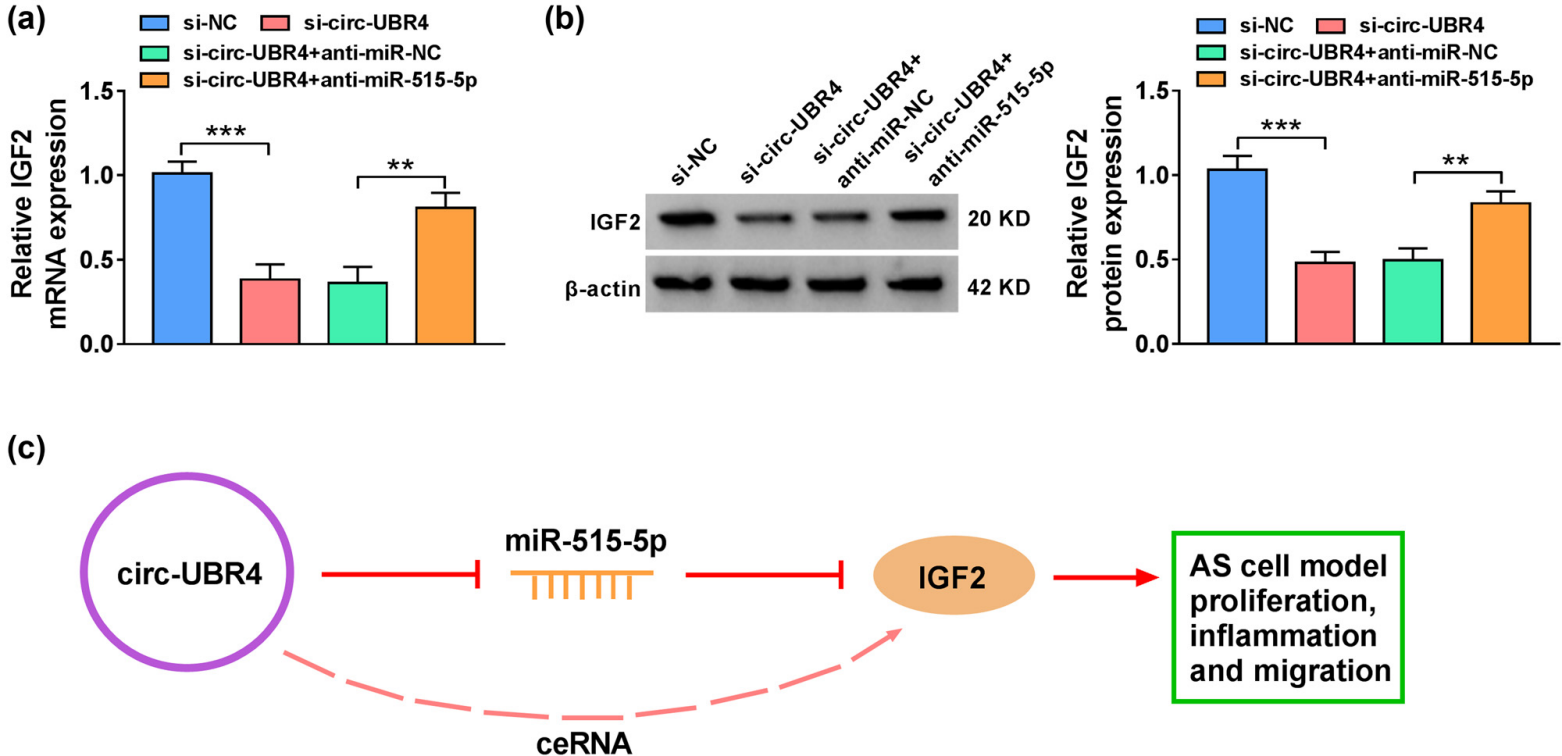
Circ-UBR4 governed the miR-515-5p/IGF2 axis. (a) and (b) The mRNA and protein expression levels of IGF2 were measured by RT-qPCR and western blot assays in VSMCs transfected with si-NC, si-circ-UBR4, si-circ-UBR4 + anti-miR-NC, or si-circ-UBR4 + anti-miR-515-5p. (c) A model for circ-UBR4/miR-515-5p/IGF2 axis was displayed. **P < 0.01, ***P < 0.001.

As shown in Figure 7a and b, circ-UBR4 knockdown decreased the expression of IGF2 in ox-LDL-stimulated VSMCs, which was recused by the silencing of miR-515-5p, at both mRNA and protein levels. To sum up, circ-UBR4 regulated IGF2 expression by targeting miR-515-5p, thereby promoting cell proliferation, inflammation, and migration in ox-LDL-stimulated VSMCs (Figure 7c).

## 4 Discussion

AS is a common risk factor for cardiovascular and cerebrovascular diseases all over the world [20]. Although majority of basic and clinical research focusing on AS, the pathogenesis mechanism of AS is not fully addressed. Our data suggested that the suppression of circ-UBR4 decreased proliferation, migration, and inflammation, while it increased the apoptosis in ox-LDL-stimulated VSMCs by targeting the miR-515-5p/IGF2 axis.

As we all know, ox-LDL could stimulate migration and proliferation of VSMCs [21]. Under pathological conditions, migrated and proliferated VSMCs in intimal layer of artery were found to participate in the early AS formation [22]. Besides, it was identified that ox-LDL stimulated cell adhesion molecules expression and thus induced a series of pathological changes, including inflammatory reactions and injury [23]. Therefore, ox-LDL-induced VSMCs were widely used as cell models of AS. Not surprisingly, inflammatory reaction was also associated with the pathogenesis of AS [24]. The pro-inflammatory cytokines, including TNF-α and IL-6, could induce dysfunction of VSMCs and promote AS progression [25]. We also confirmed that the productions of TNF-α, IL-6, and IL-1β were increased in ox-LDL-induced VSMCs.

Recently, circRNAs function as competitive endogenous RNAs to sponge miRNAs and then suppress their functions, which has been confirmed in the development of AS [26]. For example, Guo et al. reported a series of differentially expressed circRNAs in ox-LDL-induced VSMCs, including circ-UBR4 [27]. Recently, Ding et al. reported that the circ-UBR4/miR-370-3p/high mobility group box 1 networks mediated cell proliferation of ox-LDL-treated VSMCs, suggesting the important roles of circ-UBR4 in AS [10]. Our results suggested that miR-515-5p was involved in the regulatory mechanism of circ-UBR4 in AS progression.

The previous research confirmed that miR-515-5p was implicated in the development of human diseases by targeting key mRNAs, such as chromobox homolog 4 [28], IL-6 [29], and YES proto-oncogene 1 [30]. In addition, miR-515-5p was confirmed to play an important role in AS progression, and long noncoding RNA LOXL1 antisense RNA 1 could sponge miR-515-5p to facilitate the development of AS [31]. Similar to the above conclusion, we found that miR-515-5p regulated proliferation, migration, inflammation, and apoptosis of ox-LDL-treated VSMCs by targeting IGF2.

Zaina et al. provided the atherogenic activity of IGF2 in mice lacking apolipoprotein E [32]. Mechanistically, IGF2 was also involved in proliferation and apoptosis of ox-LDL-induced VSMC, and it was reported as a functional target of miR-148b [33], miR-424-5p [34], and miR-210-3p [35]. Besides, the pro-inflammation characteristic of IGF2 also was confirmed in ox-LDL-stimulate THP-1 macrophages [36]. Therefore, IGF2 served as an active participant in AS progression.

Taken together, circ-UBR4 regulated the proliferation, migration, inflammation, and apoptosis of ox-LDL-induced VSMCs via miR-515-5p/IGF2 axis. Our study enriched the role of circ-UBR4 in ox-LDL-induced VSMCs and thus provided a new perspective to understand AS pathogenesis. However, some limitations existed in our present study. We only determined the role of circ-UBR4 in cell models of AS *in vitro*, and animal models of AS were not provided in our present study. Therefore, VSMCs from various resources and animal models should be used to further validate our present findings in future work.

## 5 Conclusion

In summary, treatment with ox-LDL increased the expression of circ-UBR4 in VSMCs. We also demonstrated that the downregulation of circ-UBR4 could effectively repress proliferation, migration, and inflammation but increase apoptosis of ox-LDL-induced VSMCs via miR-515-5p/IGF2 axis, hinting that circ-UBR4 might be a new diagnostic marker for AS patients.
